# A Review of the Prospective Effects of Spacing and Varieties on Onion Yield and Yield Components (*Allium cepa* L.) in Ethiopia

**DOI:** 10.1155/2024/2795747

**Published:** 2024-03-22

**Authors:** Yohannes Gelaye, Kelemu Nakachew, Solomon Ali

**Affiliations:** ^1^Department of Horticulture, College of Agriculture and Natural Resources, Debre Markos University, P.O. Box: 269, Debre Markos, Amhara, Ethiopia; ^2^Department of Plant Science, College of Agriculture and Natural Resources, Debre Markos University, Debre Markos, Ethiopia

## Abstract

Onion (*Allium cepa* L.) is the most important commercial vegetable crop widely grown throughout the world. It is also an important bulb crop in Ethiopia. However, its production and productivity are restricted by different factors, including biotic and abiotic stresses. This review investigates the potential impacts of spacing and varieties on onion yield and yield components in Ethiopia. Countries around the world are producing onion for its nutritional value, medicinal properties, minerals, proteins, and carbohydrates. In terms of production, onion ranks second only after tomatoes. The average onion yield in Ethiopia is estimated to be 8.8 tons/ha, while in the world, it is approximately 19.7 tons/ha. Inappropriate spacing and inadequate onion varieties are some of the limitations widely described for yield variation in Ethiopia. Thus, to control the size, shape, and yield of onion bulbs, spacing determination and variety improvement are some of the techniques currently employed in Ethiopia. *Adama red*, *Bombay red,* and *red creole* are some of the known varieties in the country, and the intrarow spacings for *Adama red* and *Bombay red* are reported to be 4 cm and 6 cm, respectively. Different spacing between onion plants affects how much they produce and other factors such as size and quality, depending on the variety. It is important to assess whether changing spacing makes sense from both a farming and economic standpoint, alongside considering other agricultural methods.

## 1. Introduction

Onions (*Allium cepa* L.) are a crucial vegetable crop globally, contributing significantly to both food security and economic stability [1]. The onion is a source of proteins, carbohydrates, vitamins, and minerals. In addition, it has been described to contain *thiamine*, *riboflavin*, *niacin,* and *ascorbic* acid [2]. Thus, onion is known for its nutritional value in the human diet and medicinal properties [3].

In terms of cultivation, onion ranks second only preceded by tomatoes [4]. Globally, more than 60 million tons of onion yield are produced annually. The world yield average of onion is about 19.7 tons/ha [5]. In Ethiopia, onions are a staple crop grown by smallholder farmers across various agroecological zones. Ethiopia has immense potential to produce onion every year for both local consumption and export [6, 7]. According to the central statistical agency (*CSA*) report of 2020/21, 346,048 tons of onion yield were harvested from 38,952.58 hectares of land in Ethiopia. The average yield in Ethiopia is about 8.8 tons/ha [8]. Due to the high yield potential, ease of seed propagation, and availability of desirable cultivars, onion is known by the producers in Ethiopia [9]. In order to optimize production and ensure sustainable agricultural practices, understanding the factors influencing onion yield and yield components is essential. One of the key determinants of onion yield is the spacing between plants, which influences resource allocation, light interception, and airflow within the crop canopy. In addition, the choice of onion variety plays a vital role in determining yield and quality characteristics such as bulb size, shape, and storage potential.

However, there is limited research exploring the combined effects of spacing and varieties on onion production in the Ethiopian context [10]. Also, most small-scale farmers in Ethiopia are using traditional practices to grow onions. Therefore, improper agronomic practices, cost of production input, lack of adequate storage facilities, limited access to improved seeds, high cost of transport, fluctuation of the market price and lack of sufficient capital, and postharvest losses are among the limitations that contribute to the low yield of onion in Ethiopia [11]. Therefore, this review thoroughly investigates the potential impacts of spacing and varieties on onion yield and yield components in Ethiopia.

## 2. Methodology

In the course of conducting a literature review, the author employed various strategies. Reputable journals from the Scopus, Web of Science, and PubMed databases were utilized for the compilation of this review. In addition, the inclusion criteria primarily focused on articles published after 2019, with the exception of relevant facts and books.

## 3. Botany of Onions

*Allium cepa*, including shallots, has a diploid chromosome number (2*n* = 16), which differs significantly in storage organs such as foliage, leaves, flowering time, color, and opening order. Onions are being cultivated for bulbs and inflorescences that have been closely adapted to temperatures and photoperiods [12].

*Allium cepa* is one of the edible species of the large genus *Allium* consisting of more than 700 species [13]. Among edible species, onion (*Allium cepa* L.) is described in the first rank in the warm-temperate regions of eastern *Nepal*, followed by the group of garlic (*Allium sativum*) and shallot (*Allium cepa aggregatum*) group [14].

## 4. Importance and Production Dynamics of Onions

Onion, a major commercial vegetable crop worldwide, is known for its fungicidal, bacterial, anticholesterol, anticancer, antioxidant, and lachrymatic agents [15]. In addition, it has been documented to contain abundant phytochemicals, particularly flavonols, known for their medicinal properties [16].

**Figure 1:**
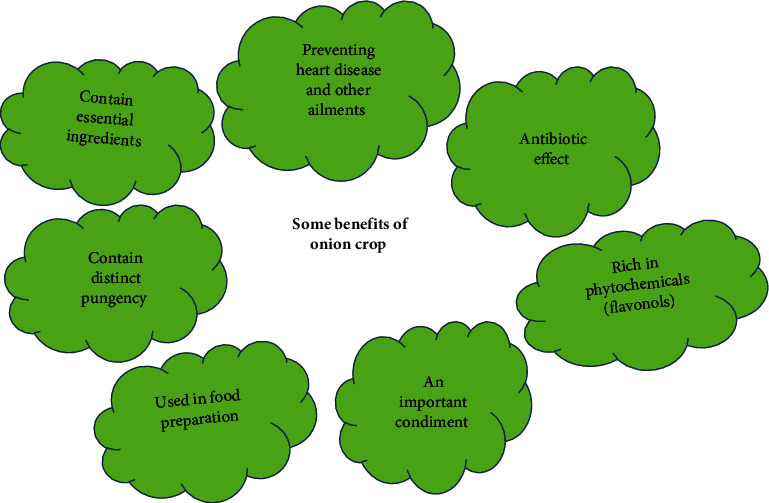
Spectacular benefits of onion crops (short summary).

Onion, a versatile condiment consumed year-round in households, utilizes both its green leaves and bulbs, whether raw or in vegetable preparation sounds like a succinct description of the usage and versatility of onions [17]. Onions are useful ingredients used in soups, sauces, seasonings, and pickled in vinegar and are valued for their distinct pungency and essential role in flavoring various dishes, sandwiches, and snacks (Figure 1).

Onions in the diet can play a role in the prevention of heart disease and other ailments [18]. *Allium*s are characterized by the presence of sulfur-containing compounds, which provide smell and pungency [19, 20]. Onion pungency develops when the alliinase enzyme interacts with sulfur-containing amino acids, such as *S-methyl -L- cysteine sulfoxide*, *S-propenyl-L-cysteine sulfoxide,* and *S-propyl-L-cysteine sulfoxide,* when cutting or crushing of onion tissue [21]. Onion flavor comes from the breakdown of compounds like pyruvate, ammonia, and sulfenic acid, leading to thiosulfinates [22, 23].

Onions are an important bulb crop in Ethiopia and were introduced to the country before 53 years and quickly became popular among producers and consumers [24]. In the early 1970s, foreigners introduced onions to Ethiopia, despite shallots being a traditional crop, and recently, onions have gained popularity and become more widely cultivated in the country [6].

Onions are a popular vegetable globally, consumed in small amounts and used daily at home in many countries [25].

Global production primarily relies on irrigation, with increasing awareness of its significance for income, food security, industry, raw materials, and employment opportunities [26]. The expansion of water harvesting structures in small-scale farmers has been reported to contribute significantly to the progress of onion production [27].

The onion crop's success relies on advanced technology, agronomic practices, and postharvest techniques, despite its uniqueness [28].

## 5. Major Characteristics of Onion

### 5.1. Agronomic characteristics of Onion Crop

Onion plants, with their shallow fibrous root system, anchor themselves in soil, absorbing water and nutrients for growth, enabling them to thrive in sandy, loamy, and clay soils alike [29]. The crop has few lateral roots, and its growth is sparse and not especially aggressive [30]. Thus, in monoculture, onion is reported to tolerate crowding, particularly in loose and friable soils [31]. The competition of weeds from aggressive root systems severely limits onion growth [32]. Onions are versatile crops that can thrive in a wide range of soil types. From sandy loam to heavy clay, as long as the soil is well draining and not waterlogged, onions can adapt and grow successfully. In Ethiopia, where the agricultural conditions vary widely, onions can be grown in different soil types, provided they receive adequate water, sunlight, and nutrients [28]. However, it is preferable to prefer well-drained sandy loam with a high content of organic matter. Onions thrive in well-drained and fertile soils with a pH between 6.0 and 7.5. They prefer full sunlight and moderate temperatures and are typically grown as cool-season crops. Due to the accumulation of soilborne diseases, onion is reported to be rotated with nonrelated crops such as beans and cereals [33]. Onions can be planted at an interval of 3 to 4 years [34]. The best growing altitude and temperature reported for onion cultivation are between 700 and 2200 ma.s.l and 15°C and 23°C, respectively [29].

Performing the right cultural practices is crucial in onion production, with typically 2-3 cultural operations recommended per growing period [35]. Cultivation is conducted three times after transplantation, at 15, 30, and 50 days, to loosen the soil around the crop's root zone [36].

With adequate irrigation and rigorous disease and pest management, regions with suitable climates can achieve year-round onion bulb production. Consideration of factors such as temperature, day length, and soil conditions is vital, along with implementing proper crop management practices such as variety selection and fertilization, to ensure successful year-round cultivation [29]. However, due to the various climatic conditions prevailing in the production areas, the yield and quality of the bulbs seem to vary from season to season. For example, the findings of the research carried out by the Melkassa Agricultural Research Center in the *upper Awash Rift Valley in Ethiopia* reported that 20 cm between rows and 10 cm between plants gave the highest yield (15 tons/ha) of onion [37].

Indeed, while onion varieties may show less pronounced differences in root and shoot systems compared to some plants, variations in growth habits, bulb size, shape, and foliage can still affect their development [10]. For example, *Bombay* red variety is said to be planted 5 cm between plants and 20 cm space between rows, and *Adama* red is spaced 6–8 cm between plants [38].

Managing onion cultivation encompasses tackling a myriad of obstacles, such as pest and disease control, weed management, water regulation, soil fertility and nutrient upkeep, climate conditions, bulb care and storage, market volatility, and labor expenses [39]. Regular monitoring and integrated strategies are necessary to manage pests and diseases such as thrips, onion maggots, downy mildew, white rot, and botrytis [40]. Effective onion growth hinges on water management, soil testing, and fertilization, while profitability is subject to market shifts and labor expenses, necessitating strategic planning and innovative agricultural approaches [41].

### 5.2. Varietal Attributes of Onion

Various onion cultivars exist globally, categorized as fresh, bunching, or dehydrator types [42]. Open-pollinated hybrid fresh market cultivars, prized for their pungency, dominate over bunching and dehydrator types [43]. Dehydrated onion mix, crafted from flax, onion powder, and rings, serves as a key ingredient in snack-making [44].

Onion cultivars are reported to vary in vegetative characteristics such as leaf length, arrangement and color [45]. Cultivars differ in bulb traits such as structure, shape, color, flavor intensity, storage capacity, size, dryness, sugar content, and maturity [46]. The dry bulb with high dry matter is cited to be firm and hence more resistant to harm [47]. Onion genotypes vary in seed stalk development ease and exhibit physiological defects such as splitting, double bulbs, thick necks, and premature bolting [42]. Onions are reported to be a highly cross-pollinated vegetable crop [48]. In Ethiopia, onion bulbs yield 3–12 flower stalks, each bearing 250–1000 flowers per umbel, with pollination rates varying from 30% to 90% based on pollinator availability [38].

Onions' distinct qualities demand meticulous selection, disease control, and monitoring by growers for harvest prediction, storage management, and consumer preference adaptation [49].

## 6. Onion Growth and Yield: Variety and Row Spacing Effects

Onion growth and yield depend on variety and spacing, impacting plant height, leaf count, length, root growth, bulb size, weight, and consistency [50]. The ideal spacing varies with variety, soil fertility, and factors such as plant density and canopy closure. For onions, spacing is determined by variety and bulb size. Large bulbs need 4–6 inches apart in 12–18-inch rows, while green onions or scallions should be closer but not overcrowded [51]. Optimal spacing is vital for plant growth by maximizing moisture, light, space, and nutrients [52]. The high yield increment is indicated with the density of the plant density up to a certain limit [53–55].

Research findings have shown that population density significantly affects the growth and yield of onions [56]. The highest yield was reported from the spacing of 20 × 10 cm [57]. The study found that onion growth and yield significantly affect onion growth and yield, with a higher percentage of small and medium bulbs obtained in narrow spacing [58]. Single row spacing in onion fields allows plants to grow without crowding, maximizing sunlight, airflow, and nutrient access. Double row spacing improves airflow and sunlight penetration and simplifies tasks such as weeding and harvesting. It reduces humidity, promotes healthier growth, and streamlines navigation in the field. The highest recorded yield (29.97 tons/ha) came from Bombay red variety planted in double rows (40 × 20 × 10 cm) [59]. The highest marketable bulb yield (26.41 tons/ha) was obtained from single row spacing (20 × 10 cm) [60]. Competition arises when multiple plants thrive in an environment, surpassing the availability of essential resources necessary for their growth and development [61]. Spacing is often reported as a limiting factor but in reality encompasses two or more of the factors already listed [56, 62]. In extreme isolation, a crop's solitary yield indicates the maximum potential per plant [63]. Reduced plant numbers, large woody plants for consumption, and aggressive weed growth can decrease yield per hectare [64].

Over time, fluctuating competitive pressures on crops can impact their population density. In scenarios of intense competition, plants in less favorable positions may suffer and ultimately face extinction [65]. This could be seen as seed waste, but it is a necessary loss to achieve the desired plant population. As population grows, plant competition rises, leading to reduced yield per plant [66]. Nevertheless, there is an upward trend in the yield per unit of area until it reaches its maximum capacity. This phenomenon is facilitated by the effective harnessing and utilization of light within the crop canopy by larger populations, even though individual plants may not attain their maximum potential [67].

Higher plant densities influence the allocation of assimilation, leading to an increased proportion of vegetative growth and a decreased proportion of reproductive output per individual plant [68]. The optimal plant population is crucial for crops with a parabolic yield response, as partitioning away from economic portions can lead to a decline in yield [69]. Suboptimal populations may trigger compensatory growth, prompting plants to compete for limited resources and resulting in a flat response and unclear population definition [70].

Optimal plant populations balance yield, quality, and profit while maximizing light interception, photosynthesis, and resource allocation for efficient production [71]. Crop canopy management often involves adjusting row spacing and plant population. As plant density rises, yield per unit area is said to reach a maximum level and eventually decline, while yield per plant typically decreases with higher plant density [72].

Onion yield increases with plant population, as canopy closure reduces weed competition. However, the yield decreases beyond an optimal limit [73]. Field trials establish the ideal plant density for various crops, accounting for factors such as weather, soil type, variety, ecology, and crop size. However, these optimal densities might not be suitable for all locations [74]. Consequently, to escape nutrient competition, adequate spacing between plants and rows is reported to be vital to obtain the maximum yield in a given plot of land [75]. Proper plant spacing is crucial for farmers to control density and maintain crop yield. Nearby plants affect individual plant environments, increasing competition and potentially changing bulb growth [76]. For example, sparse onion plants show the tallest and longest leaves, with larger bulbs and greater average weight [37]. Optimal plant density boosts bulb yield, while higher densities decrease onion leaf count due to intensified competition for nutrients and moisture [77]. Variation in the number of leaves is stated due to the genetic variation in cultivars [78]. Onion varieties are reported to vary significantly with respect to the length of leaves [79]. The research findings indicate that variations in onion population densities exert a substantial influence on several key factors including leaf number, leaf area, bulb diameter, and the yield per plant of bulbs [80].

Cultivar performance fluctuates based on agroclimatic conditions and within the same environment due to the interplay of genetic makeup and environmental factors, resulting in variations in leaf length and plant density [79]. The lowest plant density (20 plants/m^2^) was reported to give the highest leaf length (37.987 cm) followed by 30 plants (35.440 cm) [81]. This might be ascribed to increased competition for nutrients and moisture at higher plant density.

The compactness of a plant significantly influences its biomass, and adjusting the spacing between plants can serve as a useful indicator for estimating crop biomass, whether at lower or higher densities [82].

The taste of onions is affected by their sharpness and sugar levels, which correlate with the amount of dry matter present. This can differ depending on whether the onions are short-day or long-day varieties [83]. Pungency and dry matter content are important quality attributes in onions for processing [32]. Dry matter, pungency, and storability show a positive correlation, while dry matter content and bulb size exhibit a negative correlation [84]. High-yield cultivars tend to have lower soluble solid content than low-yield cultivars [85]. Moreover, a negative correlation between bulb yield and soluble solid content has also been reported [86].

The high dry matter content of onions is reported to require dehydration and better storage quality [87, 88].

Bolting is a physiological issue that harms bulb growth, and varieties vary in bolting and seed setting [89]. Higher bolting percentages occur when there are ample nutrients and space, fostering robust growth and flower stalk formation [45].

Furthermore, when planted closer together, the formation of smaller bulbs occurs, which are more resistant to bolting and can increase proportionally with the growth of the plant [84, 90, 91].

Bolting can differ because of genetics, temperature shifts, soil quality, farming methods, day length, spacing, and seedling size [92, 93].

## 7. Review Gaps

Research on onion production in Ethiopia needs to cover various aspects such as spacing, variety selection, and their impacts on yield, quality, and resilience to climatic challenges. Comprehensive variety trials assessing different onion varieties under various spacing regimes are lacking. There is a need for research to establish optimal spacing distances to maximize yield and quality while minimizing resource inputs. In addition, studies should focus on yield components, nutrient uptake, disease and pest resistance, and economic viability. Addressing these gaps can lead to sustainable onion production practices and enhance food security and farmer livelihoods in Ethiopia.

## 8. Summary and Conclusions

This study examined the effect of spacing and varieties on onion yield and yield components. It was found that inappropriate spacing and inadequate varieties were contributing factors to low onion yields in Ethiopia. Popular varieties like Adama red, Bombay red, and red creole were identified, with varying optimal intrarow spacings. The combination of 4 cm spacing and the Bombay red variety yielded the highest marketable yield. The findings highlight the importance of considering spacing, varieties, and other management practices for improving onion yield and economic outcomes.

In conclusion, this study underscores the significant influence of spacing and varieties on onion yield and yield components. It emphasizes the need for agronomic and economic considerations when determining spacing, crop varieties, and management practices. Further research should focus on cross-location experiences to validate findings and identify adaptable varieties suitable for specific spacing conditions. Continuous evaluation and identification of suitable varieties with optimal spacing are essential for enhancing onion production and addressing yield constraints in Ethiopia.

## Data Availability

Data sharing is not applicable to this article, as no new data was analyzed in this study.
